# Target blood pressure management during cardiopulmonary bypass improves lactate levels after cardiac surgery: a randomized controlled trial

**DOI:** 10.1186/s12871-021-01537-w

**Published:** 2021-12-08

**Authors:** Qing Miao, Dong Jin Wu, Xu Chen, Meiying Xu, Lin Sun, Zhen Guo, Bin He, Jingxiang Wu

**Affiliations:** 1 Department of Anesthesiology, Shanghai Chest Hospital, Shanghai Jiao Tong University, No. 241 West Huaihai Road, Shanghai, 200030 China; 2 Department of Cardiopulmonary Bypass, Shanghai Chest Hospital, Shanghai Jiao Tong University, Shanghai, China; 3 Department of Intensive Care Unit, Shanghai Chest Hospital, Shanghai Jiao Tong University, Shanghai, China

**Keywords:** Target blood pressure, Cardiopulmonary bypass, Mean arterial pressure, Lactate, Cardiac surgery

## Abstract

**Background:**

Hyperlactatemia is associated with a poor prognosis in cardiac surgery patients. This study explored the impact of target blood pressure management during cardiopulmonary bypass (CPB) on blood lactate levels after cardiac surgery.

**Methods:**

Adult patients undergoing cardiac valve surgery between 20/1/2020 and 30/6/2020 at Shanghai Chest Hospital were enrolled. The patients were randomized into a low mean arterial pressure (L-MAP) group (target MAP between 50 and 60 mmHg) or a high mean arterial pressure (H-MAP) group (target MAP between 70 and 80 mmHg), *n* = 20 for each. Norepinephrine was titrated only during CPB to maintain MAP at the target level. Blood lactate levels in the two groups were detected before the operation (T0), at the end of CPB (T1), at the end of the operation (T2), 1 h after the operation (T3), 6 h after the operation (T4) and 24 h after the operation (T5). The primary outcome was the blood lactate level at the end of the operation (T2). The secondary outcomes included the blood lactate level at T1, T3, T4, and T5 and the dose of epinephrine and dopamine within 24 h after the operation, time to extubation, length of stay in the ICU, incidence of readmission within 30 days, and mortality within 1 year.

**Results:**

Forty patents were enrolled and analyzed in the study. The lactate level in the H-MAP group was significantly lower than that in the L-MAP group at the end of the operation (3.1 [IQR 2.1, 5.0] vs. 2.1 [IQR 1.7, 2.9], *P =* 0.008*)* and at the end of CPB and 1 hour after surgery. The dose of epinephrine within 24 h after the operation, time to extubation and length of stay in the ICU in the L-MAP group were significantly higher than those in the H-MAP group.

**Conclusions:**

Maintaining a relatively higher MAP during CPB deceased the blood lactate level at the end of surgery, reduced epinephrine consumption, and shortened the time to extubation and length of stay in the ICU after surgery.

**Trial registration:**

This single-center, prospective, RCT has completed the registration of the Chinese Clinical Trial Center at 8/1/2020 with the registration number ChiCTR2000028941. It was conducted from 20/1/2020 to 30/6/2020 as a single, blinded trial in Shanghai Chest Hospital.

## Background

Hyperlactatemia usually occurs after cardiac surgery, which is related to poor prognosis [1, 2] and is reported to be an independent risk factor for postoperative mortality in cardiac surgery patients [3]. Therefore, alleviating postoperative hyperlacticaemia is vitally important for improving the prognosis of cardiac patients [4].

Blood flow has a pulsatile characteristic in normal heart activities. However, nonphysiological advection perfusion is adopted during cardiopulmonary bypass (CPB) in many heart centers. Unlike advection perfusion, which provides kinetic energy to normalize blood flow, pulsatile perfusion reduces the potential energy under the same mean arterial pressure (MAP), resulting in decreased blood flow in capillaries and microcirculation perfusion. Thus, we supposed that a relatively high MAP during CPB may benefit microcirculation perfusion in reducing blood lactate levels, which is a sensitive indicator of poor microcirculation perfusion. Based on the Guidelines for CPB in 2019 European Adult Cardiac Surgery [5], it is safe to maintain the MAP between 50 and 80 mmHg during CPB. However, in clinical practice, we observed a clinical phenomenon in which a relatively higher MAP within the recommended range during CPB seemed to be correlated with a lower postoperative blood lactate level. To verify this phenomenon, we conducted a randomized controlled trial (RCT) to test the primary hypothesis that mean arterial pressure maintained at 70–80 mmHg by norepinephrine titration [6] during CPB could reduce the lactate level after cardiac surgery and improve the early recovery of patients.

## Methods

### Clinical data

This single-center, prospective, RCT study was conducted in accordance with the Declaration of Helsinki tenets and the Basel, and approved by the Shanghai Chest Hospital of Shanghai Jiaotong University research ethics board (KS1960) at 31/10/2019 and registered at the Chinese Clinical Trial Center on 8/1/2020 with registration number ChiCTR2000028941. This study was performed by adhering to the Consolidated Standards of Reporting Trials (CONSORT) statement. Forty patients who were scheduled for cardiac valve surgery in the Cardiovascular Surgery Department of Shanghai Chest Hospital (Shanghai, China) from 20/1/2020 to 30/6/2020 were enrolled in the study. All enrolled patients signed their informed consent without knowing their groups. The inclusion criteria were patients older than 18 years with a New York Heart Association Class (NYHAC) level II-III, blood lactate level < 1.6 mM•L^− 1^, alanine aminotransferase (ALT) < 50 U•L^− 1^, creatinine (Cr) < 111 ųM•L^− 1^, blood urea nitrogen (BUN) < 9.5 mM•L^− 1^, and brain natriuretic peptide (BNP) < 100 pg•ml^− 1^ who required observation in the intense care unit (ICU) after surgery. The ICU researchers were blinded to the group assignments. The exclusion criteria were patients who underwent reoperation within 24 h after the initial operation due to surgical factors.

### Interventions and measurements

The enrolled patients were randomized into a low mean arterial pressure (L-MAP) group (target MAP between 50 and 60 mmHg) and a high mean arterial pressure (H-MAP) group (target MAP between 70 and 80 mmHg) based on the random numbers generated by EXCEL software in a 1:1 ratio. The randomization sequence was generated by staff not otherwise involved in the current study. MAP in the L-MAP group was maintained in the range of 50–60 mmHg according to the clinical experience of the anesthesiologist. A bolus of phenylephrine (0.25–0.5 mg per injection, maximum dose < 2 mg) was given when the MAP was under 50 mmHg. Infusion of nitroglycerin (0.2 ~ 4 μg/kg/min) was started when the MAP was over 60 mmHg during CPB. MAP was managed at a target of 70–80 mmHg in the H-MAP group by norepinephrine titration [initial rate: 0.03 ~ 0.1 μg/kg/min, maximum rate: 0.4 μg•(kg•min)^− 1^] during CPB. MAP was continuously measured every 15 min and used for analysis in both study groups. The area under or upper the curve (AUC) out of the target blood pressure range was calculated as previously described [7]. MAP was maintained over 65 mmHg postoperatively in both groups. Norepinephrine (0.03 ~ 0.1 μg/kg/min), nitroglycerin (0.2 ~ 4 μg/kg/min), epinephrine (0.03 ~ 0.1μg/kg/min) and dopamine (3 ~ 10μg/kg/min) were used if *necessary* according to clinical experience or routine clinical practice after the operation. The blood lactate level in the two groups was measured before the operation (T0), at the end of CPB (T1), at the end of the operation (T2), 1 h after the operation (T3), 6 h after the operation (T4) and 24 h after the operation (T5). The cumulative dosage of the anesthetic and vasoactive drugs, heart rate (HR), hematocrit (HCT), blood glucose, Cr, and BUN in the perioperative period, duration of surgery, duration of aortic occlusion, duration of CPB, time to the end of surgery after weaning from CPB, time to extubation, length of stay in the ICU, incidence of readmission within 30 days and one-year all-cause mortality were recorded and compared.

### Anesthesia and CPB

Patients in the two groups were treated with the same protocol anesthesia method and CPB management. Radial arterial cannulation and arterial blood gas analysis were carried out before anesthesia induction. General anesthesia was induced with 0.8 μg•kg^− 1^ sufentanil, 0.12 mg•kg^− 1^ midazolam, 2 mg•kg^− 1^ propofol and 0.15 mg•kg^− 1^ cis-atracurium intravenously. Drugs were target controlled and infused with propofol in 4–8 mg•(kg•h)^− 1^, remifentanil in 0.1–0.15 μg•(kg•h)^− 1^ and cis-atracurium in 0.1–0.3 mg•(kg•h)^− 1^ in the maintenance period. All anesthetic medications were stopped at the end of the surgery. All patients underwent CPB based on the Guidelines for CPB in 2019 European Adult Cardiac Surgery [5]. After splitting the sternum, CPB was established through the ascending aorta and superior and inferior vena cava. Cardioplegia was perfused through the aortic root or coronary opening. CPB was prefilled with 1000 ml hydroxyethyl starch and 500 ml lactated Ringer. CPB started after systemic heparinization and ACT > 480 s. Both groups were on occlusive CPB. The perfusion flow was maintained at 2.4 L•min^− 1^•m^− 2^, and the mixed venous blood oxygen saturation was maintained above 75% under mild hypothermia conditions. Intraoperative blood glucose was controlled to 8 mM•L^− 1^ by insulin infusion. The ascending aorta was cross-clamped followed by warm blood cardioplegia perfusion through the aortic root or coronary opening when the nasopharyngeal temperature was 32–34 °C. After the operation, rewarming, ascending aorta recovery, cardiac rebeating and auxiliary circulation were carried out until hemodynamics became stable and the nasopharyngeal temperature reached 36.8 °C. CPB was stopped, and protamine was injected to neutralize heparin. The patient was sent to the ICU for observation after surgery.

### Study endpoints

The primary outcome was the blood lactate level at the end of surgery (T2). The secondary outcomes were blood lactate levels at the end of CPB (T1), T3, T4, and T5; dose of epinephrine and dopamine within 24 h after surgery; time to extubation; length of stay in the ICU; readmission within 30 days; and mortality within 1 year.

### Statistical analysis

Continuous variables including age, body mass index (BMI), left ventricular ejection fraction (LVEF), clinical data and blood lactate levels are presented as the mean ± standard deviation (SD), median [first quartile, third quartile] or N (N/total number of patients%). Kolmogorov–Smirnov tests were used to assess the distribution of continuous variables. Normally distributed variables were analyzed using Student’s t test; otherwise, nonnormally distributed variables were analyzed using Mann–Whitney U-tests. Classified variables, including sex, ASA classification, hypertension, diabetes prevalence, NYHAC and surgical type, were recorded as numbers or percentages. Categorical variables were compared using a chi-square test or Fisher’s exact test as appropriate.

The blood lactate level and MAP at multiple time points were analyzed with ANOVA for repeated measures and least significant difference post hoc testing. *P* < 0.05 (2-sided) was considered statistically significant. We estimated the effect of continuous monitoring on the area under or upper the curve (AUC) out of target blood pressure using the 2-sample Wilcoxon rank-sum test and Hodges Lehmann estimation of location shift with the corresponding asymptotic 95% CI. The balance of baseline and surgical characteristics was compared using absolute standardized difference scores (ASDs) as previously described [8]. Imbalance was defined as ASD (Cohen’s d) greater than 0.20 (small effect size), 0.5 (median effect size) and 0.8 (large effect size). Considering that the sample size of the current study was small, ASD > 0.50 was selected as an indication of potential confounding and to adjust for such factors directly in the analyses comparing the groups on the outcome.

### Sample size calculation

The sample size was calculated based on our pre-experimental results. The lactate level in the H-MAP group was 2.46 ± 1.13 mM•L^− 1^ vs. 3.68 ± 1.16 mM•L^− 1^ in the L-MAP group at the end of the operation. It was calculated that 20 cases for each group were needed based on α = 0.05 and β = 0.1 using a two-sided two-sample unequal-variance t test.

## Results

### Baseline characteristics

The basic characteristics of the enrolled patients are summarized in Table 1. There were no significant differences in age, sex, BMI, ASA classification, hypertension, diabetes prevalence, LVEF NYHAC classification, or surgical type. Between the two groups.

**Table 1:** Baseline characteristics of the patients

Characteristics	L-MAP Group (*n* = 20)	H-MAP Group (*n* = 20)	ASD^a^
Age (yr)	61 [45,68]	63 [53,69]	0.12
Sex (M/F)	12/8	8/12	0.45
BMI (kg•m^− 2^)	25.4 [20.0, 26.4]	21.9 [20.6, 26.4]	0.16
ASA (II/III)	5/15	3/17	0.35
Hypertension, n (%)	7 (35)	8 (40)	0.12
Diabetes prevalence, n (%)	1 (5)	1 (5)	0.00
LVEF (%)	60 [50, 63]	63 [61, 65]	0.44
NYHAC (II/III)	7/13	12/8	0.56
Surgical types, n (%)			0.12
AVR	1 (5)	5 (25)	
AVP	2 (10)	1 (5)	
MVR	3 (15)	1 (5)	
MVP	2 (10)	1 (5)	
TVP	0 (0)	2 (10)	
AVP + MVP	2 (10)	0 (0)	
AVR + MVR + TVP	0 (0)	2 (10)	
AVR + MVP + TVP	4 (20)	2 (10)	
MVR + TVP	5 (25)	1 (5)	
MVP + TVP	1 (5)	5 (25)	
Operation time (min)	270 [240, 300]	260 [210, 280]	0.55
Aortic occlusion time (min)	90 [82, 127]	110 [80, 132]	0.19
CPB time (min)	140 [120,160]	150 [110, 170]	0.07
Post CPB time (min)	84 [63, 95]	70 [64, 76]	0.51

*Abbreviations*: *MAP* Mean arterial pressure, *BMI* Body mass index, *ASA* American Society of Anesthesiologists, *LVEF* Left ventricular ejection fraction, *NYHAC* New York Heart Association. *AVR* Aortic valve replacement, *AVP* Aortic valvuloplasty, *MVR* Mitral valve replacement, MVP Mitral valvuloplasty, *TVP* Tricuspid valvuloplasty. ^a^*ASD* Absolute standardized difference = difference in means or proportions divided by the standard error; imbalance defined as an absolute value greater than 0.20 (small effect size), 0.5 (median effect size) and 0.8 (large effect size)

**Fig. 1:**
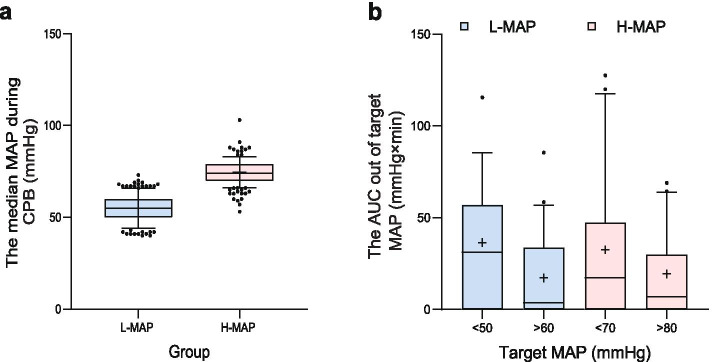
The median MAP and the time out of the target MAP during CPB

The MAPs during CPB are shown in Fig. 1. Figure 1a is a box & whiskers plot with 10–90 percentile of the median MAP during CPB of the two groups. The median MAP in each group was successfully maintained in their target blood pressure range (57 mmHg in L-MAP vs. 76 mmHg in H-MAP). Figure 1b. shows the AUC out of the target MAP. There was no significant difference between the two groups.

### Blood lactate level

**Fig. 2:**
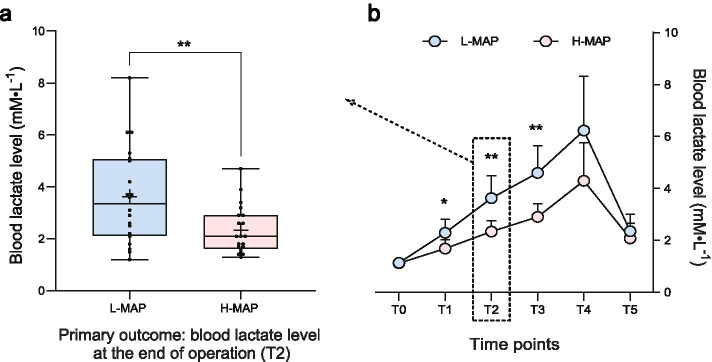
Blood lactate level. *Statistically significant (*P < 0.05 **P < 0.01)). Abbreviations: MAP: mean arterial pressure. T0 = before the operation; T1 = at the end of CPB, T2 = at the end of the operation, T3 = 1 h after the operation, T4 = 6 h after the operation, T5 = 24 h after the operation

As shown in Fig. 2, the lactate level in the H-MAP group was significantly lower than that in the L-MAP group at the end of surgery. The lactate levels of the two groups began to show differences from the end of CPB until 1 hour after surgery. After reaching the peak at 6 h after the operation, the lactate level began to drop, but there were no differences between the two groups (Fig. 2b). The primary outcome was the blood lactate level at the end of surgery (T2), presented in Fig. 2a as a box & whiskers plot with min to max, showing all points.

### Clinical data

As shown in Table 2, there were no statistically significant differences between the two groups in parameters such as HR, HCT, blood glucose, Cr, perioperative BUN, duration of surgery, duration of aortic occlusion, CPB and time to the end of surgery after weaning from CPB, readmission rate within 30 days, and mortality within 1 year. There was also no significant difference in anesthetic and vasopressor usage between the two groups. The cumulative postoperative epinephrine administered within 24 h, the time of extubation and the length of stay in the ICU in the L-MAP group were significantly higher than those in the H-MAP group.

**Table 2:** Perioperative clinical data

Clinical Data	L-MAP Group (*n* = 20)	H-MAP Group (n = 20)	*P* Value
Hemodynamics parameter			
HR (bpm)			
baseline	79 [72, 86]	81 [75, 85]	0.321
30 min post CPB	88 [78, 92]	87 [83, 90]	0.827
at the end of the operation	90 [83, 97]	87 [80, 98]	0.147
MAP (mmHg)			
before induction	87 [73, 98]	92 [83, 110]	0.698
30 min post CPB	67 [60, 70]	70 [68, 73]	0.697
end of the operation	67 [63, 77]	70 [63, 73]	0.638
1 h after the operation	68 [60,76]	71 [68, 77]	0.072
6 h after the operation	71 [70, 79]	68 [64, 72]	0.543
24 h after the operation	75 [70, 79]	70 [61, 75]	0.646
Perioperative medications			
Anesthetics			
Propofol (mg)	2683.2 [2080,2878.4]	2142.4 [1820, 2534.4]	0.457
Sufentanil (μg)	56.2 [48, 63.4]	52.8 [49.4, 63.4]	0.607
Midazolam (mg)	8.4 [7.2, 9.5]	7.9 [7.4, 9.5]	0.521
Cis-atracurium (mg)	67.1 [52, 72]	58.6 [45.5, 79.1]	0.472
Remifentanil (μg)	33.5 [26, 36]	29.3 [22.8, 39.6]	0.416
Vasopressor drugs			
Phenylephrine during CPB (mg)	0.35 [0.15, 0.48]	0.4 [0.3, 0.52]	0.854
Norepinephrine during CPB (mg)	0.17 [0, 0.19]	0.72 [0.47, 0.95]	0.000***
Epinephrine within 24 h after the operation (mg)	0.72 [0, 1.8]	0 [0, 0]	0.024*
Dopamine within 24 h after the operation (mg)	314 [0, 420]	0 [0, 300]	0.088
Vasodilator drugs			
Nitroglycerin during CPB (mg)	1.4 [0, 2.1]	0 [0, 2.2]	0.000***
Nitroglycerin within 24 h after the operation (mg)	0 [0, 0.5]	0 [0, 0.7]	0.243
Laboratory parameters			
HCT (%)			
during CPB	26.8 [24.2, 29.8]	25.9 [25.2, 28.8]	0.892
at end of surgery	29.8 [29.2, 34.8]	28.9 [28.7, 33.8]	0.317
Blood glucose during CPB (mM•L^−1^)	8.4 [7.3, 9.6]	8.6 [6.9, 9.2]	0.874
Cr (ųM•L^−1^)			
Baseline	78 [63, 90]	74 [60, 84]	0.558
6 h after the operation	72 [58.8, 88]	79 [52, 93]	0.690
24 h after the operation	75 [57, 91]	75 [63, 86]	0.706
BUN (mM•L^−1^)			
Baseline	6.6 [5.3, 7.1]	6.0 [5.0, 6.8]	0.774
6 h after the operation	6.9 [5.1, 7.8]	6.3 [5.2, 7.4]	0.928
24 h after the operation	7.9 [6.4, 9.8]	7.5 [5.1, 9.8]	0.935
Urine volume (ml)			
during CPB	300 [200, 600]	600 [300, 800]	0.086
24 h after the operation	2855 [2700, 3130]	3010 [2410, 3330]	0.589
Outcome data			
Extubation time (h)	17 [17,18]	11 [6,16]	0.042*
Length of stay in the ICU (h)	43 [42, 68]	36 [19, 43]	0.008**
Readmission within 30 days (n)	0	0	null
Mortality in 1 year (%)	0	0	null

*Statistically significant (*P < 0.05 **P < 0.01***P < 0.001). Norepinephrine and nitroglycerin during CPB are interventional factors for target blood pressure management. *Abbreviations*: *CPB* Cardiopulmonary bypass, *MAP* Mean arterial pressure, *HCT* Hematocrit, *ICU* Intensive care unit

## Discussion

The cause of hyperlactacidemia falls into two types: insufficient perfusion or hypoxia of the tissue, such as shock or ischemia [4], and activation of the pyruvate replacement pathway due to underlying diseases, such as liver diseases, malignant tumors, or mitochondrial myopathy [9]. Insufficient perfusion or hypoxia occurs widely during CPB after cardiac surgery, which forms the focus of this study. Most currently available guidelines for the treatment of severe septic shock patients recommend the use of norepinephrine to increase MAP above 65 mmHg when the volume therapy loses effectiveness, which is believed to be beneficial to improving circulatory perfusion and lactic acidosis [10]. Therefore, sufficient MAP is the main controllable factor to ensure microcirculation perfusion. It is common knowledge that the lactate level directly reflects the perfusion condition and is closely related to the prognosis of patients. Thus, we hypothesized that organ perfusion could be improved by appropriately adjusting MAP during CPB, although the perfusion flow is constant.

In this study, we observed the impact of targeted blood pressure management during CPB on lactate levels after cardiac surgery and found that the blood lactate level in the H-MAP group was significantly lower than that in the L-MAP group at the end of surgery and 1 h after surgery, suggesting that maintaining a relatively higher perfusion pressure (MAP 70–80 mmHg) during CPB was more conducive to improving the perfusion and oxygen supply to reduce lactate levels after surgery. This conclusion was further confirmed by the shortened time of extubation, reduced length of ICU stay, and use of less epinephrine for circulation support 24 h after the operation in the H-MAP group.

In addition, MAP before anesthesia induction showed no significant difference between the two groups. Although studies have shown that blood pressure before anesthesia induction does not reflect the actual blood pressure of patients [11], maintaining the MAP close to the level before anesthesia induction during CPB will benefit patients. Knowing that hyperlactacidemia during CPB is closely related to postoperative mortality in cardiac surgery, it is important to redress the risk factors for hyperlactacidemia [12], which is defined as a perioperative blood lactate level above 3 mM•L^-^ 1[13]. In both groups, blood lactate reached the level of hyperlactacidemia at the end of surgery and at 6 h after surgery. The lactate level in the H-MAP group was significantly lower than that in the L-MAP group. Similar results were shown in the study of Siepe [14], but their target MAP was 80–90 mmHg for H-MAP vs. 60–70 mmHg for L-MAP, which was higher than that in our study. GOLD believed that a higher MAP (80–100 mmHg) during CPB was safe and effectively improved outcomes after coronary bypass [15]. However, in Vedel’s study, it was reported that a low level of MAP does not modify lactate during cardiac surgery [6], but the operation time, bypass time, and cross clamp time were all shorter than those in our research. This means that the longer surgery time, bypass time, and cross clamp time may be related to the postoperative lactate level. Compared with research showing that a high level of MAP during normothermic CPB does not reduce the risk of postoperative AKI and there is no difference in the peak of lactate postoperation [16], our study yielded different results for postoperative lactate levels when the temperature was 32–34 °C during CPB; this finding also implied that temperature correlates with lactate levels during the perioperative period. It has been reported that oxygen delivery inadequate to fulfill the metabolic needs of the patient is one of the factors promoting hyperlactatemia during CPB, which is associated with reactive hyperglycemia that is probably due to insulin resistance triggered by catecholamine release [1]. In our study, blood glucose was controlled to 8 mM•L^− 1^ by insulin infusion during the perioperative period. Considering these factors, we further compared HR, MAP, HCT, blood glucose, Cr, BUN, surgery time, aortic occlusion time, CPB time and post-CPB time, finding no significant differences between the two groups.

Most current studies on how to maintain a safer MAP during CPB focus on the relationship between MAP and postoperative acute kidney injury (AKI) or cognitive dysfunction. Although there was no significant difference in postoperative cognitive dysfunction between the H-MAP group (70–80 mmHg) and the L-MAP group (40–50 mmHg) [17], maintaining a relatively high MAP level during CPB could effectively reduce the occurrence of AKI [18]. However, there were no significant differences in Cr, BUN and urine volume between the two groups in this study, mainly because we targeted a MAP between 50 and 60 mmHg in the L-MAP group instead of 40–50 mmHg. This result is consistent with the conclusion that maintaining a high MAP during CPB could improve perfusion.

We found that certain individual differences mattered much in target blood pressure management. Preoperative basic blood pressure and the antihypertensive drugs used by the patients should all be taken into consideration when performing blood pressure management precisely. On the other hand, many vasoconstrictors, such as epinephrine, could potentially bias the lactate value perioperatively. It has been reported that the median dose of epinephrine increased plasma lactate by 0.25 μg/kg/min, while dopamine had the opposite effect [19]. Norepinephrine usually does not induce an increase in the plasma lactate concentration [20]. In this study, vasoconstrictors were used only when necessary, and the dose of epinephrine used was less than 0.25 μg/kg/min and had little effect on our results.

There are also some limitations in this study. For instance, this is a single center with a limited number of cases. Due to the small sample size, we compared the baseline of patients by absolute standardized difference scores (ASDs) [8]. We found that the ASDs of the two groups for the NYHAC (II/III), operation time, and post-CPB time were more than 0.5, which indicated an imbalance between the two groups and may have introduced bias in the results. However, the ASDs of ASA (II/III) and LVEF (%) were less than 0.5, and the ASDs of the aortic occlusion time and CPB time were less than 0.2. Considering all of these factors, it was believed that such potential confounding factors may not have affected the results directly. Furthermore, all blood lancet levels increased significantly from post CPB until 6 h after the operation and were even more than 3 mM•L^− 1^ during that time. Therefore, multicenter, larger-sample studies are required in the future. Based on the finding that a relatively high MAP improved tissue perfusion during CPB, our future study will monitor the peripheral circulation during CPB by ultrasound and calculate oxygen supply and consumption for more convincing evidence [21, 22].

## Conclusion

Targeted MAP management between 70 and 80 mmHg during CPB could better ameliorate the lactate level after cardiac surgery compared with MAPs between 50 and 60 mmHg. A relatively high blood pressure may be more conducive to improving tissue perfusion and oxygen supply, reducing the lactate level, and shortening the time of extubation and length of ICU stay, all of which are beneficial to cardiac surgery patients.

## Data Availability

The datasets used and/or analyzed during the current study are available from the corresponding author on reasonable request.

## Abbreviations

CPB: Cardiopulmonary bypass
ICU: Intense care unit
NYHAC: New York Heart Association Class
BMI: Body mass index
LVEF: Left ventricular ejection fraction
ASA: American Society of Anesthesiologists
HR: Heart rate
HCT: Hematocrit
Cr: Creatinine
BUN: Blood urea nitrogen
MAP: Mean arterial pressure
AVR: Aortic valve replacement
AVP: Aortic valvuloplasty
MVR: Mitral valve replacement
MVP: Mitral valvuloplasty
TVP: Tricuspid valvuloplasty
